# Off-Stoichiometry Thiol–Enes Polymers Containing Silane Groups for Advanced Packaging Technologies

**DOI:** 10.3390/polym14101988

**Published:** 2022-05-13

**Authors:** Kirill Puchnin, Dmitriy Ryazantsev, Vitaliy Grudtsov, Yaroslav Golubev, Alexander Kuznetsov

**Affiliations:** 1 Scientific-Manufacturing Complex “Technological Centre”, Zelenograd 124498, Russia; d.ryazancev@tcen.ru (D.R.); v.grudtsov@tcen.ru (V.G.); kae@tcen.ru (A.K.); 2 Institute of Nanotechnology of Microelectronics of the Russian Academy of Sciences, Moscow 119991, Russia; 3 A.V.Topchiev Institute of Petrochemical Synthesis, RAS, Moscow 119991, Russia; ya_golubev@ips.ac.ru

**Keywords:** OSTE, silane, thiol–ene, photocuring, bonding, microfluidic, packaging, integration

## Abstract

New modified off-stoichiometry thiol–enes polymers, called OSTE-MS polymers, were developed by introducing mercaptosilane into the polymer mixture. This modification made it possible to introduce silane groups into the polymer frame, due to which the polymer gained the ability to bond with silicon wafers without modification of the wafer surface by any adhesive. The optimal composition for creating 3D polymer structures on a chip was selected, which consists of a volume ratio of 6:6:1 of allyl monomer, mercapto monomer, and mercaptosilane, respectively. The hardness, shift force, tensile strength, Young’s modulus, optical transparency, glass transition temperature, thermal stability, and chemical resistance of the OSTE-MS polymer, and the viscosity for the prepolymer mixture were studied. On the basis of the OSTE-MS polymer, 3D polymer structures of the well type and microfluidic system on the silicon chips were obtained.

## 1. Introduction

In 2011, a new polydimethylsiloxane (PDMS) replacement material, off-stoichiometry thiol–enes (OSTEs) polymers, was proposed to simplify the transition from research prototyping to commercial production of microfluidic devices. This polymer consists of two monomers, one containing three or four thiol functional groups and the other containing three allyl functional groups [1]. The uniqueness of this material is in the ability to customize mechanical properties, such as the Young’s modulus (E) and the glass transition temperature (T_g_), as well as direct UV-bonding of polymer pieces [2]. In addition, such polymers can be bonded to a silicon surface. To bond OSTE polymers with silicon wafers, vinyl silane or 3-isocyanatopropyl triethoxysilane (IPTES) is immobilized on the wafer surface. When the polymer comes into contact with the wafer, the excess of mercapto groups of the polymer come into a “click” reaction with the terminal groups of the silane. In the case of vinyl silane, the reaction occurs with UV initiation, and, in the case of IPTES, the “click” reaction occurs spontaneously [3].

A further development of OSTE polymers is the introduction of different additives into the polymer composition. Such polymers are called OSTE+ polymers [4]. For instance, the addition of polyethylene glycol to prepolymer composition leads to an increase in polymer hydrophilicity [5]; the addition of an imidazole fragment makes it possible to obtain materials for selective and efficient removal of gold from aqueous media [6], and the most common modification of OSTE+ polymers is the injection of epoxy, for example, bisphenol A diglycidyl ether. The OSTE+ polymers with epoxy can be processed via dual-cure. The first stage of this process is the UV-initiated interaction of mercapto and vinyl groups, and the second stage is the interaction of mercapto and epoxy groups during heating. This interaction leads to changes in the mechanical properties of the polymer. In addition, epoxy groups have the ability to interact with surface hydroxyl groups, which are used to bond the polymer to silicon surfaces [4].

It should be particularly noted that OSTE polymers can be easily further modified by using a thiol–ene “click” reaction due to the presence of an excess of mercapto or allyl groups in the polymer. So, by using 3,3,4,4,5,5,6,6,7,7,8,8,9,9,10,10-heptadecafluorodecylmethacrylate and 2-hydroxyethyl methacrylate, one is able to obtain a hydrophobic or hydrophilic polymer surface, respectively. [7] Varying the chemical modifier, various surface functional groups such as hydroxyl, carboxylic acid, amine, fluorine, imidazole, epoxide, PEG, and zwitterionic groups are obtained. [8,9]. In addition, aptamers [10] and various enzymes, such as xylanas [11], horseradish peroxidase [12], α-chymotrypsin [13], or glucose oxidase [14,15], can be immobilized on the polymer surface.

As of today, OSTE polymers are widely used in applied research. In addition to common microfluidic systems, OSTE polymers are used for various applications: nanoliter well arrays for liquid storage [16], optical tweezers [17], synthetic paper used in immunoassays [18], devices for plasma separation [19], the chips capable of sample desalting, concentration and separation [20], or enzymatic digestion prior to mass spectrometry [21], substrates for flexible electrodes [22], and gate dielectric for use in organic thin-film transistors (OTFTs) [23]. Composite materials have also been developed based on OSTE polymers. The addition of superparamagnetic carbonyl iron particles to OSTE made it possible to obtain tunable and magnetic micropillar arrays [24]. Encapsulation of an organometal hybrid perovskite nanocrystal in an OSTE polymer reduces photodegradation [25] and increases the photoluminescence bandwidth [26]. An OSTE polymer matrix for silicon quantum dots in a luminescent solar concentrator improves Si nanocrystal quantum yield [27]. Thus, OSTE polymers are promising and are already widely used in research.

Despite the fact that many organosilicon-related materials have been developed to date [28], OSTE polymers containing silane fragments are not represented in the literature. In this work, we have developed OSTE polymers that include silane groups. They combine the advantages of OSTE polymers and the ability of silanes to excellently bond to silicon oxide surfaces; so, these polymers can be bonded to the silicon surface without a preliminary stage of surface modification. This modification of the polymer expands the possibility of using such polymers in modern technologies. In the first part of our study, the optimization of the polymer composition is considered; in the second, the physicochemical properties of the optimized polymer are studied; and in the third, the practical application of the developed polymer is demonstrated.

## 2. Materials and Methods

### 2.1. Materials

All chemicals received from commercial sources were used without further purification. Pentaerythritol tetrakis(mercaptoacetate) (PETMA, 90%, CAS: 10193-99-4), triallyl isocyanurate (TATATO, 98%, CAS: 1025-15-6), and 2,4,6-trimethylbenzoylphenylphosphinic acid ethyl ester (TPO-L, 95%, CAS: 84434-11-7) were purchased from ABCR. (3-Mercaptopropyl)trimethoxysilane (MS, 98%, CAS: 4420-74-0) was purchased from Sigma-Aldrich (St. Louis, MO, USA). The P-type silicon-on-insulator (SOI) wafers were purchased from Shanghai Simgui Technology Co. (Shanghai, China). Chips were manufactured according to the 1.2 μm CMOS technology by the Scientific and Manufacture Complex “Technological center” (Zelenograd, Russia). Solvents were purified and dried according to standard procedures.

### 2.2. OSTE-MS Preparation

The ratio of TATATO, PETMA, or MS was varied when selecting the polymer composition. For the general preparation method for optimized OSTE-MS polymer, 2 mL TATATO, 2 mL PETMA, 0.33 mL MS, and 0.02 mL TPO-L were mixed. Mixing was carried out at room temperature by using the rotator at 30 rpm for 15 min. Liquid prepolymer was poured into a mold. The sample was irradiated with UV LED (365 nm, 10 W) for 1 min per 1 mm thickness of the OSTE-MS polymer layer. After that, the sample was heated at 110 °C for 15 min. If the samples needed to be bonded to the surface, about 0.5 MPa pressure was applied to them during the heating stage.

### 2.3. Characterization

The Fourier transform-infrared (FT-IR) spectrum was recorded on a Nicolet iS50 FT-IR with a diamond crystal attenuated total reflection (ATR) accessory (Thermo Scientific, Waltham, MA, USA). A total of 64 scans per measurement was used.

NMR specter was acquired at 25 °C on AVANCE 400 (Bruker, Bremen, Germany) spectrometer. Extraction was carried out from an OSTE-MS polymer sample weighing 30 mg with 0.7 mL of CDCl_3_ for 1 h. A total of 32 scans per measurement was used.

The hardness of polymers was measured on the Digital Shore Durometer Type D (XINGWEIQIANG) with the use of a mechanical stand. Polymer cubes 9.5 × 9.5 × 9.5 mm in size were used for measurements. The result was recorded after 15 s of contact between the durometer and the polymer. The final result was taken from the average of three measurements.

The shift force of the polymeric columns was measured by using the digital force gauge AMF-200 (ALIYIQI Instrument, Beijing, China). The OSTE-MS polymer columns 4 mm high and 1.65 × 1.65 mm wide were made on a silicon wafer pretreated with UV/ozone (BioForce Nanosciences, Ames, IA, USA). A force parallel to the plane of the wafer was applied to the columns. The shift force was equal to the maximum force at which the column came off the wafer. The final result was taken from the average of at least three measurements.

The tensile strain of the OSTE-MS polymers under tension was measured on an AGS-10 (Shimadzu, Kyoto, Japan) universal testing machine at room temperature. The samples were cast in a 3-D printed PLA mold. The test speed was 2 mm/min. The final result was taken from the average of three measurements.

UV-visible absorption spectra were recorded on the Infinite M200pro (Tecan, Männedorf, Switzerland) spectrophotometer by using trUView Cuvettes (Bio-Rad Laboratories, Hercules, CA, USA). The prepolymer mixture was subjected to polymerization directly in the cuvette.

The thermophysical characteristics of the polymer were measured on a thermogravimetric analyzer and differential scanning calorimeter combined instrument STA 449 F3 Jupiter (NETZSCH, Selb, Germany). The weight of the samples was approximately 11 mg. The measurements were carried out in the temperature range from 23 °C to 700 °C at a heating rate of 1 °C/min and 10 °C/min. Air was used as the reaction gas. Alumina crucibles were used.

The viscosity of the prepolymer mixture was measured on a hybrid rheometer Discovery HR-1 (TA Instruments, New Castle, DE, USA). The prepolymer mixture was used in the measurements without the addition of a UV initiator TPO-L. A 20-mm parallel plate was used for geometry. A gap of 1 mm was used.

To measure the chemical resistance, polymer cubes 9.5 × 9.5 × 9.5 mm in size were used. The chemical resistance was evaluated by three parameters: hardness, geometric dimensions, and weight. Parameters were measured after one hour, one day, and one week of conditioning. According to the totality of changes in these parameters, the solvents were divided into four nominal groups: good, normal, satisfactory, and unsatisfactory chemical resistance. Full details of parameter changes are presented in the Supplementary Materials Tables S1–S3.

### 2.4. Formation of Polymer Structures on a Chip

A Vaseline-wax sacrificial layer was applied to the silicon chips by using a Nordson EFD Ultimus V Fluid Dispenser. After formation of the sacrificial structure, it was filled with the prepolymer, irradiated with ultraviolet light, put under pressure, and heated for 15 min at 110 °C. The sacrificial layer was removed by double sonication with white spirit and double wash using hexane. The “well” type structure consists of four walls connected to each other in the form of a rectangle. The microfluidic structure is parallelepiped containing two cylindrical holes for tube connection, which are interconnected by a microchannel.

## 3. Results and Discussion

### 3.1. Preparation of OSTE-MS Polymers

Monomers that were previously used in the literature to create off-stoichiometry thiol–ene polymers (OSTE), pentaerythritol tetrakis(mercaptoacetate) (PETMA), and triallyl isocyanurate (TATATO) were chosen to obtain the new polymers with additives (OSTE+) [7]. Silanes containing a thiol or vinyl terminal group are suitable for including silane groups into the polymer. These groups have the ability to click-interact with the corresponding groups of monomers, embedding into the polymer framework during photopolymerization (Figure S1). Different silanes have different reactivity [29]. For instance, trichlorosilanes can easily undergo hydrolysis in air, which leads to the instability of the adhesion properties of the polymers containing trichlorosilane groups. In addition, trichlorosilanes, when hydrolyzed, emit hydrochloric acid, which leads to corrosion that may be problematic for some approaches. Trialkoxysilanes are devoid of these disadvantages. They are also hydrolyzed in air but at a slower rate than trichlorosilanes, and when trialkoxysilanes are hydrolyzed, alcohol is formed, which easily evaporates without harming the microchips. Moreover, the modifier of OSTE+ must be dissolved in a mixture of TATATO and PETMA. (3-Mercaptopropyl)trimethoxysilane (MS) has all of the above properties; so, it was chosen as the polymer modifier, which was abbreviated as OSTE-MS.

**Figure 1:**
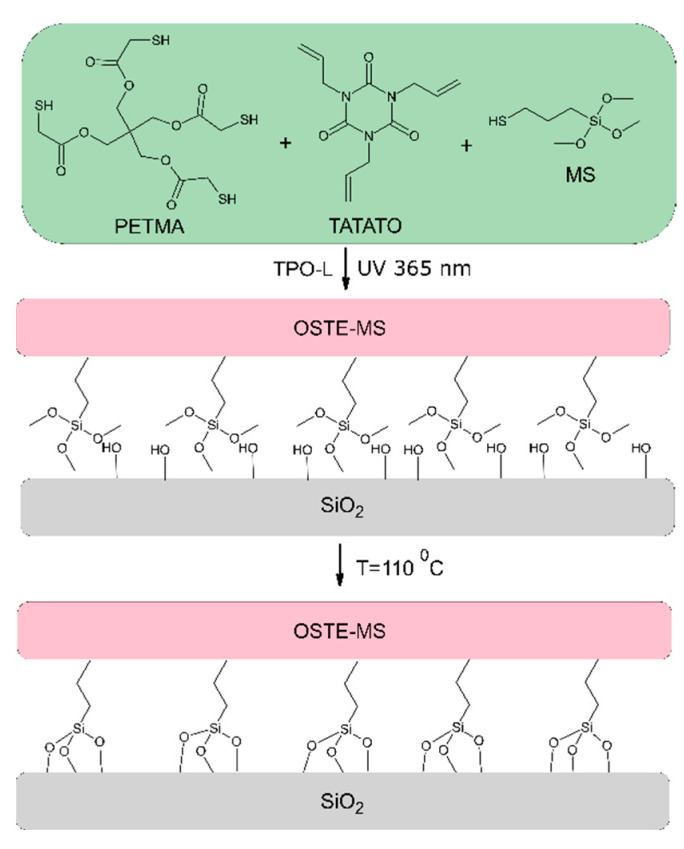
Schematic illustration of the OSTE-MS polymer fabrication process and the crosslinking with the surface.

The process of creating OSTE-MS polymer structures on a chip consists of two steps: photopolymerization occurring due to ultraviolet radiation and crosslinking—the interaction of the polymer silane groups with surface hydroxy groups during heating (Figure 1). During the photopolymerization of OSTE polymers, the mixture self-heats [30], which can cause the premature activation of silanes. It affects the second step of OSTE-MS polymer structure creation, worsening polymer adhesion. Therefore, it is necessary to avoid overheating the mixture during photopolymerization. For this, in some cases, we used a gradual increase in UV irradiation power, which led to less heating of the sample.

To initiate the silane groups, the samples were heated. Under these conditions, they can interact with surface hydroxyl groups [29]. For the best contact during polymer bonding with the silicon surface, a pressure of 0.5 MPa was applied. Higher pressure led to the destruction of the polymer at the heating stage. To obtain identical physicochemical properties, polymer samples that did not need to be bonded to the surface were also subjected to heating.

**Figure 2:**
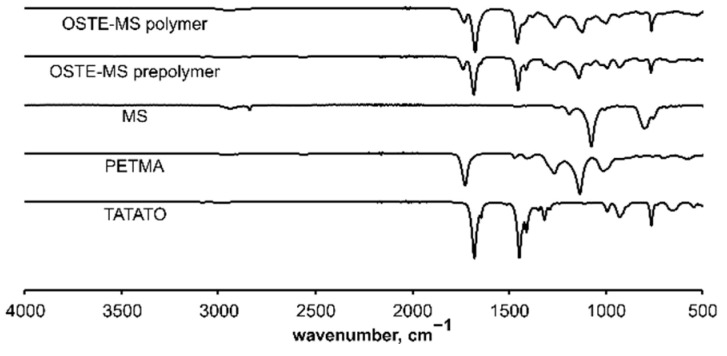
The FT-IR spectra of OSTE-MS polymer after heating 15 min, OSTE-MS prepolymer and monomers (MS, PETMA, TATATO).

The completeness of the polymerization reaction was confirmed by FT-IR spectroscopy. Because the ratio of allyl and thiol groups in the selected OSTE-MS polymer was close to equimolar, the bands at 2840 and 2570 cm^−1^ corresponding to thiols of MS and PETMA, respectively, and the band at 1644 cm^−1^ corresponding to allyl of TATATO disappeared after polymerization (Figure 2).

Studies have also been conducted to identify residues of unreacted monomers by extraction. The resulting OSTE-MS polymer was placed in CDCl_3_, kept for 1 h, after which the ^1^H NMR spectrum was recorded. There were no signals from the initial reagents in the spectrum (Figure S2), which indicates the inclusion of silanes into the polymer framework.

### 3.2. Optimization of Polymer Composition

**Figure 3:**
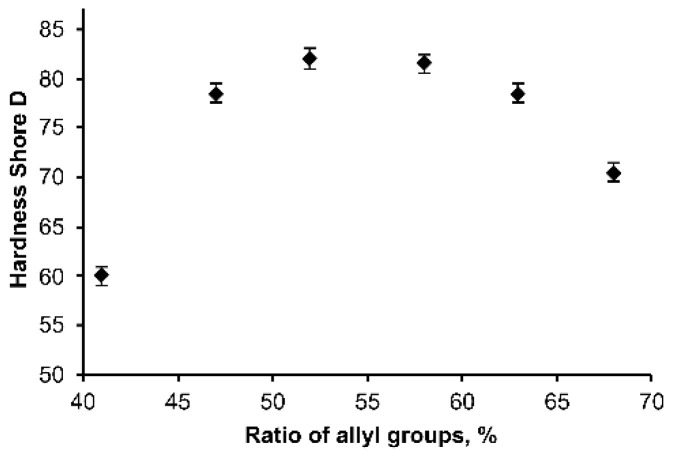
Dependence of the hardness Shore D on the ratio of allyl groups in the OSTE-MS polymers.

It is known that the ratio of monomers has a decisive role in the mechanical properties of OSTE polymers [1]; so first, the ratio of the reagents in the polymer was studied. Thus, the hardness of the OSTE-MS polymers was measured at a fixed MS content and various ratios of PETMA and TATATO. It turned out that in the range of 50–60% allyl groups content (the ratio of allyl groups to the sum of allyl and thiol groups from PETMA, TATATO, and MS, Figure 3) the polymer hardness was suitable for 3D polymeric structures formation. The highest hardness was observed at a maximum of 52% allyls, which corresponds to an equal volume ratio of PETMA and TATATO. This turned out to be very convenient for ease of operation and the elimination of human error in sample preparation. Therefore, in all further experiments, an equal volume ratio of PETMA and TATATO was used.

**Figure 4:**
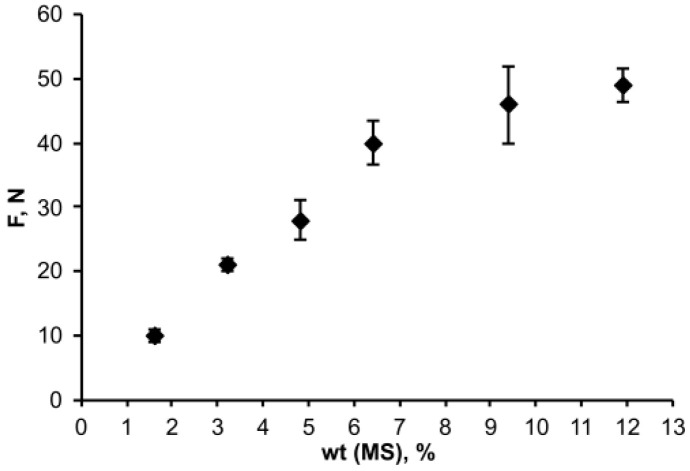
Dependence of the shift force on the concentration of silane in the OSTE-MS polymers.

At the next stage of the work, the influence of MS content in the polymer on the ability to crosslink with the silicon wafers was studied. It turned out that with an increase in the concentration of silane in the polymer, the force required for the shift detachment of the polymer column from the silicon wafers increased. Moreover, at a low concentration of silane (lower approx. 6.4 wt.%) in the polymer, an intensive increase in the shift force occurred; then at higher silane concentrations, the increase in the shift force slowed down (Figure 4).

**Figure 5:**
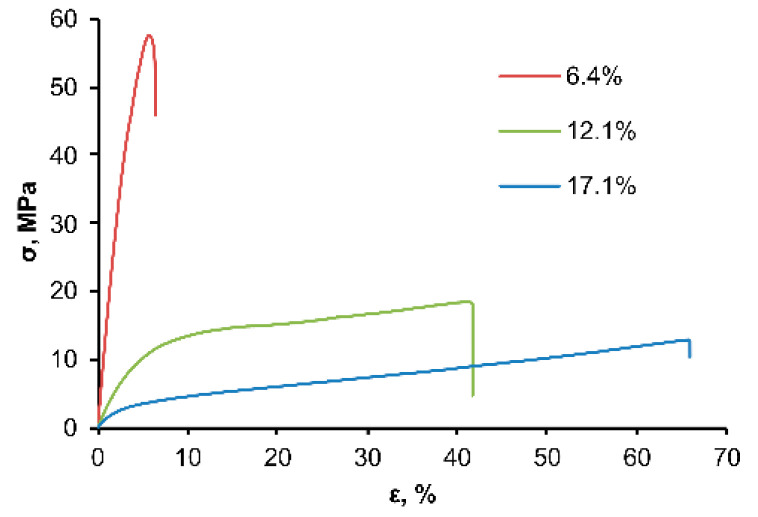
The mechanical characteristics of OSTE-MS polymers.

It turned out that the mechanical characteristics of the OSTE-MS polymers studied in this work were dramatically determined by the silane content in the polymer. With an increase in the silane concentration, there was a significant decrease in tensile strength and a decrease in Young’s modulus (Figure 5). Thus, at silane contents of 6.4, 12.1, and 17.1 wt.%, the values of tensile strength were 58, 19, and 13 MPa, and the values of Young’s modulus were 1.7, 0.4, and 0.1 GPa, respectively. Perhaps this is due to the fact that when MS interacted with TATATO, the TATATO had no more than two allyl groups, which led to an increase in linear fragments and a decrease in crosslinks (Figure S1).

Summarizing the data on shift force, tensile strength, and Young’s modulus, a composition consisting of volume ratios of TATATO, PETMA, and MS 6:6:1 was chosen, as this composition was found to be optimal for creating 3D polymer structures on a chip that was used for our lab’s applications (maximum hardness and good bonding to silicon chips). Therefore, further studies of the physicochemical properties were carried out with this ratio of reagents.

### 3.3. Physicochemical Properties of OSTE-MS Polymer

For optimized OSTE-MS polymer composition, the absorption specter was measured in the range of 230–1000 nm. The obtained OSTE-MS polymer had optical transparency in the range of 420–1000 nm but featured substantial absorption at wavelengths below 420 nm (Figure S3). These data were similar to the absorption of other OSTE polymers [30]. The absorption peak at 370 nm corresponded to the absorption of the TPO-L photoinitiator. Therefore, if it is necessary to extend the optical transparency of OSTE-MS to the near ultraviolet region, one can replace TPO-L with a photoinitiator which has a lower shortwave absorption peak.

**Figure 6:**
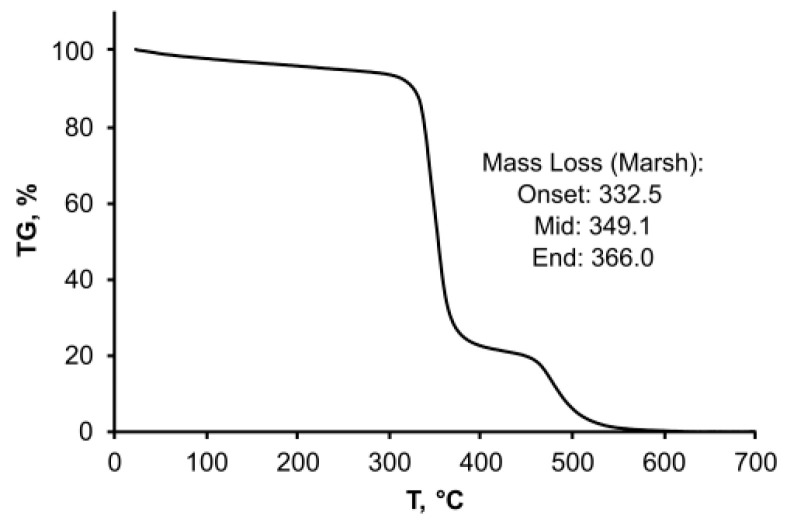
TGA curve of OSTE-MS polymer in air. Heating rate 1 °C/min.

The thermogravimetric analysis of the sample was done by using a heating rate of 1 °C/min, and demonstrated the thermal stability of the OSTE-MS polymer in the air up to 330 °C (Figure 6). This temperature was achieved due to the formation of a three-dimensional framework and the absence of short fragments in the OSTE-MS polymer. The polymer was charred during pyrolysis and started to fully decompose at temperatures over 460 °C. The onset of degradation for OSTE-MS was shifted to a lower temperature compared with the stoichiometric thiol–ene formulation from the literature [31]. We assumed that this was due to the inertia of the system, so we repeated the experiment, setting the heating rate as in the literature to 10 °C/min. Indeed, under these conditions, the bend of the TGA curve shifted to about 400 °C (Figure S4), as for the previously described the OSTE polymers. Curves repeat each other. First, the endothermic decomposition of the polymer occurs at about 400 °C, then the polymer burns out (exothermic process) at temperatures above 500 °C. However, we consider the data obtained at 1 °C/min to be more reliable.

The glass transition temperature of the OSTE-MS polymer measured by differential scanning calorimetry was 52 °C (see Figure S5). One of the important parameters for casting is the viscosity. The viscosity of the OSTE-MS prepolymer was measured at 15, 20, and 25 °C. In these analyses, a photoinitiator was not added to the mixture because during the measurement, the required UV protection could not be provided, and local polymerization would distort the obtained viscosity values. An increase in the silane concentration in the mixture of monomers led to a decrease in the viscosity of the mixture, and the temperature dependence of the viscosity decreased. For instance, the viscosity of a TATATO:PETMA mixture with a 1:1 volume ratio without MS at 20 °C was 0.66 Pa∙s; with the addition of 6.4 and 12.1 wt.% MS, it decreased to 0.34 and 0.21 Pa∙s, respectively. See the Supplementary Materials Table S4 for viscosity values for other temperatures.

Because the OSTE-MS polymer is planned to be used for packaging, its interaction with various solvents is possible; therefore, the chemical resistance to main organic solvents was investigated. Chemical resistance was assessed visually, by changes in size, weight, and hardness. We chose a change in weight less than 1%, size less than 2%, and hardness less than 2 HD as the arbitrary criterion for chemical resistance. For “Good” samples, these parameters did not go beyond the specified values within one week, while “Normal” and “Satisfactory” indicate the same for one day and one hour, respectively. For “Unsatisfactory” samples, the parameters were out of the values in less than one hour (Table 1).

The OSTE-MS polymer degraded in chloroform and methylene chloride, crumbling into small pieces (Figure S6). The polymer gradually dissolved in acetic acid, whereas its weight and size decreased, and the hardness remained almost unchanged. The polymer swelled in the remaining solvents, in which it behaved as “Normal” and “Satisfactory”. In this case, an increase in mass and size and a decrease in hardness were observed. The dynamics of the changing parameters are presented in the Supplementary Materials.

### 3.4. Practical Application

**Figure 7:**
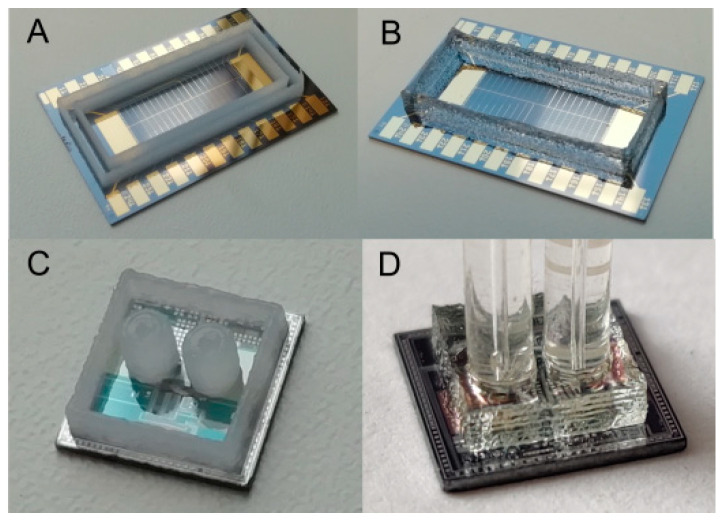
Formation of polymer structures on a silicon chip: (**A**,**C**) making of a sacrificial layer from Vaseline ink, (**B**) finished structure of the “well” type, and (**D**) a microfluidic system.

By using the developed OSTE-MS polymer, the possibility of 3D structure formation was demonstrated. Three-dimensional well-type structures and a microfluidic system on a microchip were obtained. Both of them were formed by using a sacrificial layer of Vaseline-wax ink. The OSTE-MS prepolymer was poured into the Vaseline base and photopolymerized; then, the structure was placed under pressure and heated. As a result, the polymer was crosslinked with the chip. The sacrificial layer was easily removed in white spirit (Figure 7).

The obtained samples showed good stability when working with buffer solutions and good dielectric properties. This was tested using a “well” structure with a wall in the middle. The resistance between the 0.3-mm-thick wall separating two buffer 3M KCl solutions was >1 TOhm. This makes it possible to use the developed OSTE-MS polymers as an insulator in microchips.

## 4. Conclusions

In conclusion, in this work a new OSTE+ polymer containing silane groups was developed. The optimal polymer composition for creating three-dimensional structures on silicon chips was selected, containing a 6:6:1 volume ratio of PETMA, TATATO, and MS. The Young’s modulus of such a polymer was 1.7 GPa, the tensile strength was 58 MPa, the glass transition temperature was 52 °C, and the viscosity of the prepolymer was 0.34 Pa∙s. The developed polymer was optically transparent in the range of 420–1000 nm and thermally stable up to 330 °C. A study of the chemical resistance of the OSTE-MS polymer showed good resistance to main organic solvents, except for chloroform and methylene chloride. On the basis of the OSTE-MS polymer, “well”-type 3D structures and a microfluidic system were obtained. The developed OSTE-MS polymer can be widely used in advanced sensing technologies, including its application as a photosensitive dielectric, and also as a component of the direct writing technology, in particular within the framework of the “lab-on-a-chip” concept.

## Figures and Tables

**Table 1:** The chemical resistance of OSTE-MS in organic solvents at room temperature.

Solvent	Chemical Resistance ^1^
Methanol	Good
Ethanol	Good
Hexane	Good
Decane	Good
2-Propanol	Good
Benzene	Good
Limonene	Good
Toluene	Good
Tetrachloromethane	Good
Ethyl acetate	Normal
2-Butanone	Normal
Acetic acid	Normal
Dimethyl sulfoxide	Satisfactory
Tetrahydrofuran	Satisfactory
Acetone	Satisfactory
Acetonitrile	Satisfactory
Dimethylformamide	Satisfactory
Dichloromethane	Unsatisfactory
Chloroform	Unsatisfactory

^1^ See the text for explanation of “good”, “normal”, “satisfactory”, and “unsatisfactory” chemical resistance parameters.

## Data Availability

Not applicable.

## Supplementary Materials

The following supporting information can be downloaded at: https://www.mdpi.com/article/10.3390/polym14101988/s1, Table S1: OSTE-MS polymer size change during conditioning in various solvents; Table S2: OSTE-MS polymer weight change during conditioning in various solvents; Table S3: OSTE-MS polymer hardness change during conditioning in various solvents; Figure S1. The linear fragment of OSTE-MS polymer containing a silane group; Figure S2: NMR spectrum of the extract of OSTE-MS after aging for 1 h (CDCl_3_); Figure S3: UV-light absorption spectra of OSTE-MS polymer; Figure S4: TGA curve of OSTE-MS polymer in air. Heating rate 10 °C/min; Figure S5: DSC curves of OSTE-MS polymer; and Table S4: OSTE-MS polymer viscosity at different temperatures and mercaptosilane content in the mixture; Figure S6: Photo of the OSTE-MS polymer cube before (A) and after (B) treatment in chloroform (24 h).
