# Supplementary material for: Off-Stoichiometry Thiol–Enes Polymers Containing Silane Groups for Advanced Packaging Technologies
Source: Polymers (Basel). 2022 May 13;14(10):1988. doi: 10.3390/polym14101988 (PMC9147012; doi:10.3390/polym14101988)
Supplement: Supplementary file 1 [file polymers-14-01988-s001.zip › polymers-1709018-supplementary.pdf]

Supplementary Material

Off-stoichiometry thiol–enes polymers containing silane groups for advanced packaging technologies

Kirill Puchnin*, Dmitriy Ryazantsev, Vitaliy Grudtsov, Yaroslav Golubev and Alexander Kuznetsov
puchninkv@yandex.ru

Table S1. OSTE-MS polymer size change during conditioning in various solvents

Solvent	Δl, %		
Time, h	1	24	168
Methanol	-0.3	-1.2	-1.7
Ethanol	-0.3	-0.6	-0.7
Hexane	-0.4	-0.9	-0.4
Decane	-0.5	-1.0	-0.7
2-Propanol	0.5	0.1	0.1
Benzene	-0.2	-0.3	-0.2
Limonene	0.1	-0.3	0.0
Toluene	-0.1	-1.5	-2.0
Tetrachloromethane	0.0	-0.1	-1.6
Ethyl acetate	0.0	-0.1	0.4
2-Butanone	0.1	-0.2	0.5
Acetic acid	-0.3	-0.9	-2.8
Dimethyl sulfoxide	0.8	0.9	3.8
Tetrahydrofuran	-0.4	0.4	4.0
Acetone	0.0	0.2	3.2
Acetonitrile	-0.4	1.0	4.2
Dimethylformamide	0.4	2.9	8.7
Dichloromethane	0.2	degradation	degradation
Chloroform	0.1	degradation	degradation

Table S2. OSTE-MS polymer weight change during conditioning in various solvents

Solvent	Δm, %		
Time, h	1	24	168
Methanol	0.0	0.1	0.2
Ethanol	0.1	0.1	0.1
Hexane	-0.1	0.0	0.0
Decane	-0.1	0.0	0.0
2-Propanol	0.0	0.0	0.0
Benzene	0.0	0.1	0.2
Limonene	0.1	0.2	0.3
Toluene	0.0	0.0	0.1
Tetrachloromethane	0.0	0.0	0.0
Ethyl acetate	0.1	0.5	1.1
2-Butanone	0.1	0.6	1.9
Acetic acid	0.0	0.0	-5.5

Dimethyl sulfoxide	0.5	2.5	5.7
Tetrahydrofuran	0.3	2.2	6.1
Acetone	0.4	2.3	6.4
Acetonitrile	0.8	3.8	9.3
Dimethylformamide	1.0	3.7	12.1
Dichloromethane	4.9	degradation	degradation
Chloroform	2.7	degradation	degradation

Table S3. OSTE-MS polymer hardness change during conditioning in various solvents

Solvent	Δm, %		
Time, h	1	24	168
Methanol	82.0	83.0	82.0
Ethanol	83.0	83.0	83.0
Hexane	82.0	83.5	83.5
Decane	82.0	84.0	84.0
2-Propanol	82.5	82.0	84.0
Benzene	84.0	84.0	83.0
Limonene	83.0	82.0	82.5
Toluene	83.5	83.0	82.5
Tetrachloromethane	83.5	82.0	83.5
Ethyl acetate	83.5	84.0	83.0
2-Butanone	83.0	82.5	80.0
Acetic acid	84.0	83.0	82.5
Dimethyl sulfoxide	83.5	79.5	77.0
Tetrahydrofuran	83.0	81.5	76.0
Acetone	83.0	74.5	69.0
Acetonitrile	81.0	73.0	58.0
Dimethylformamide	81.5	77.5	69.5
Dichloromethane	75.0	degradation	degradation
Chloroform	80.0	degradation	degradation

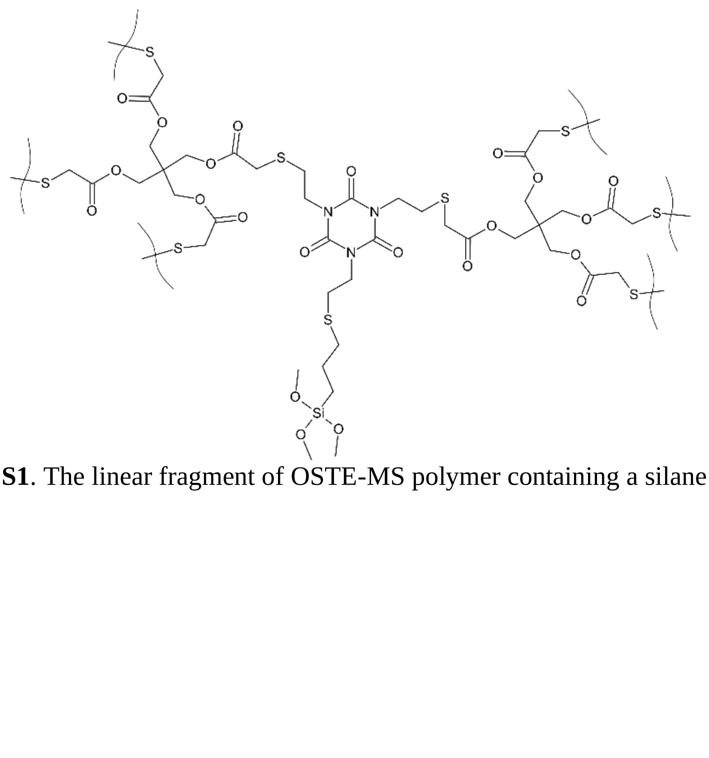

Figure S1. The linear fragment of OSTE-MS polymer containing a silane group.

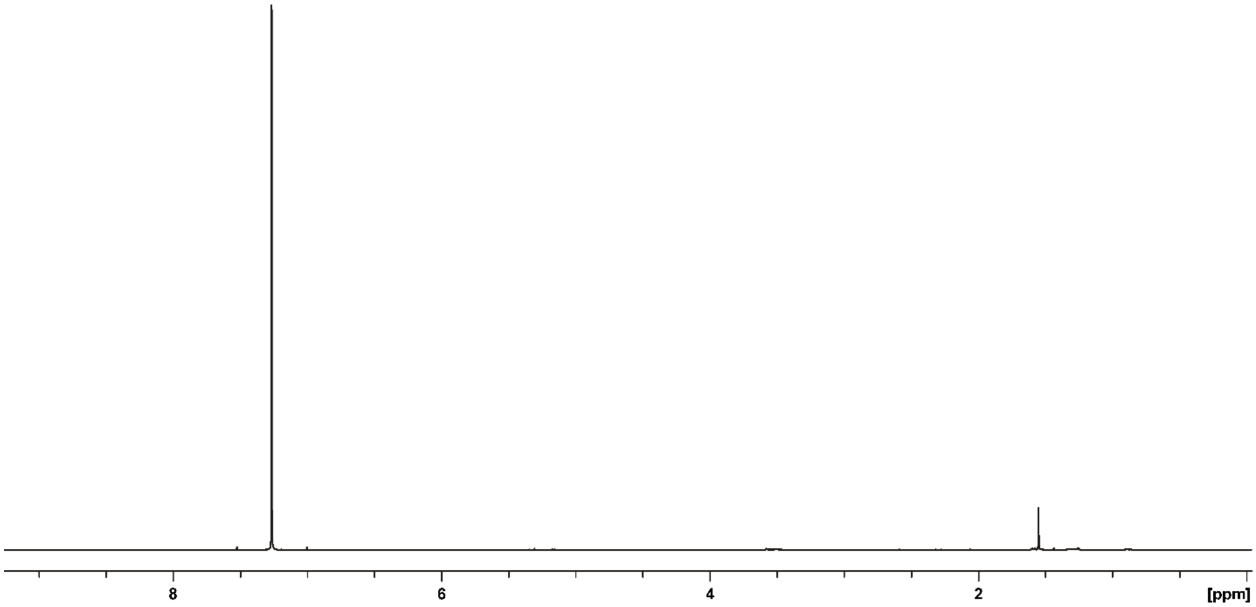

Figure S2. NMR spectrum of the extract of OSTE-MS after aging for 1 hour ($CDCl_3$).

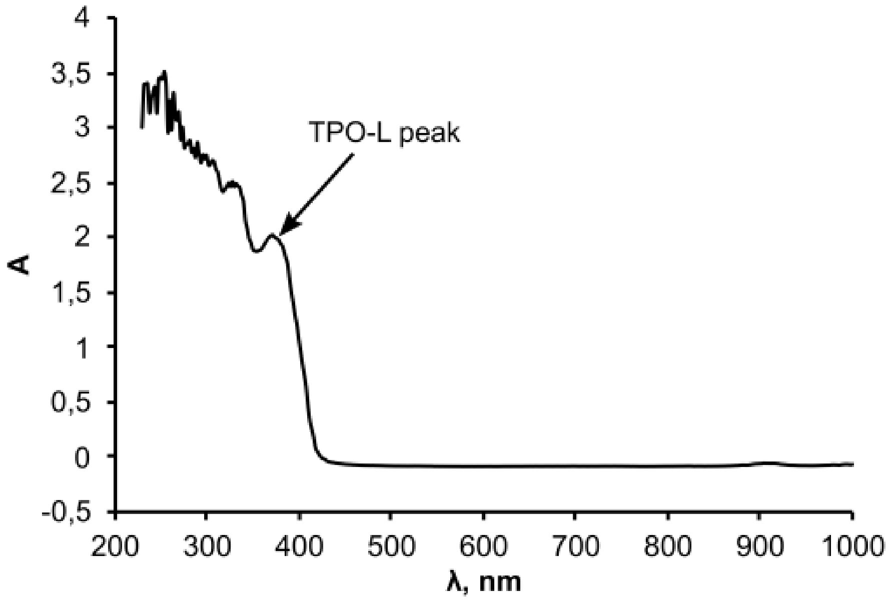

Figure S3. UV-light absorption spectra of OSTE-MS polymer.

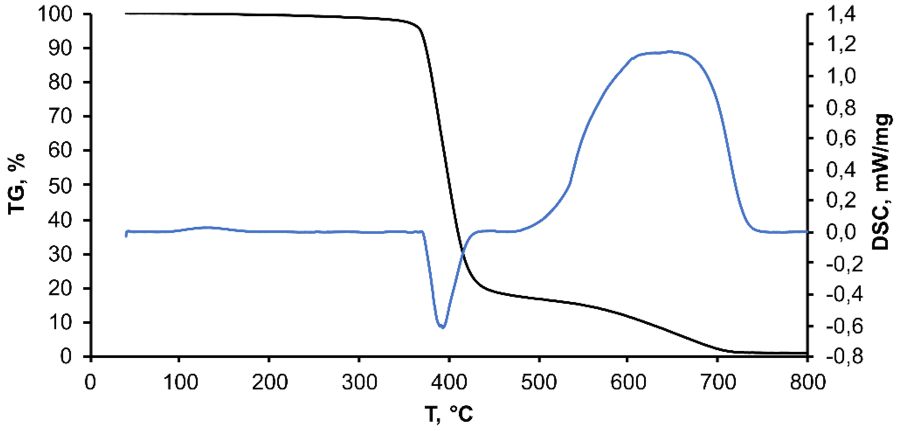

Figure S4. TGA curve of OSTE-MS polymer in air. Heating rate 10 °C/min.

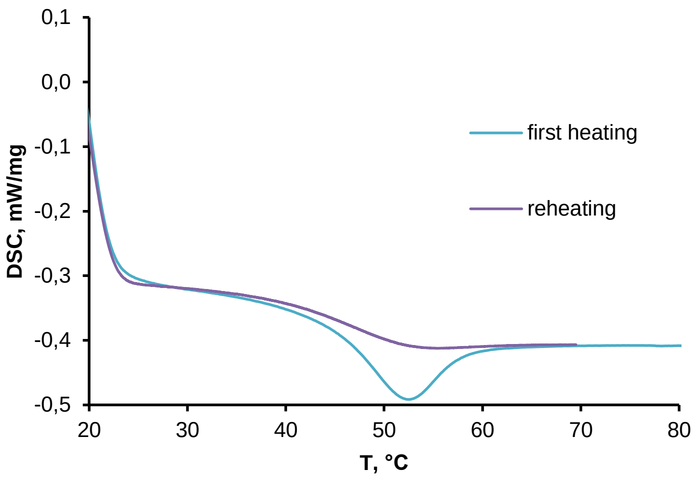

Figure S5. DSC curves of OSTE-MS polymer.

Table S4. The OSTE-MS polymer viscosity at different temperatures and mercaptosilane content in the mixture

T, °C	Viscosity, Pa·s		
wt (MS), %	0	6.4	12.1
15	0.95	0.44	0.26
20	0.66	0.34	0.21
30	0.47	0.27	0.18

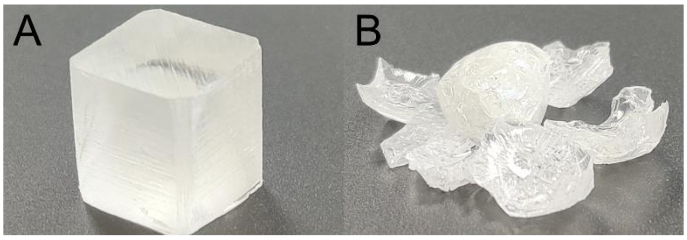

Figure S6. Photo of the OSTE-MS polymer cube before (A) and after (B) treatment in chloroform (24 hours).
